# Single-Cell Sequencing Reveals that DBI is the Key Gene and Potential Therapeutic Target in Quiescent Bladder Cancer Stem Cells

**DOI:** 10.3389/fgene.2022.904536

**Published:** 2022-06-03

**Authors:** Jiaxi Yao, Yue Liu, Jitao Yang, Mengling Li, Simin Li, Bo Zhang, Rui Yang, Yuchong Zhang, Xiaoyu Cui, ChunQing Feng

**Affiliations:** ^1^ Department of Urology, The First Affiliated Hospital of China Medical University, Shenyang, China; ^2^ Department of Medical Oncology, The First Affiliated Hospital of China Medical University, Shenyang, China; ^3^ Department of Clinical Epidemiology and Center of Evidence-Based Medicine, The First Affiliated Hospital of China Medical University, Shenyang, China; ^4^ College of Medicine and Biological Information Engineering, Northeastern University, Shenyang, China; ^5^ Key Laboratory of Data Analytics and Optimization for Smart Industry, Northeastern University, Shenyang, China; ^6^ Department of Urology Surgery, The Central Hospital Affiliated to Shenyang Medical College, Shenyang, China

**Keywords:** cancer stem cells, bladder cancer, scRNA-seq, acetaminophen, DBI

## Abstract

**Background:** Drug resistance and recurrence often develop during the treatment of muscle-invasive bladder cancer (MIBC). The existence of cancer stem cells (CSCs) in MIBC makes the formulation of effective treatment strategies extremely challenging. We aimed to use single-cell RNA sequencing approaches to identify CSCs and evaluate their molecular characteristics and to discover possible therapeutic measures.

**Methods:** GEO data sets GSE130001 and GSE146137 were used to construct an expression matrix. After cells were identified by type, malignant epithelial cells inferred by InferCNV were extracted for stemness evaluation. The subset of cells with the highest stemness was subjected to weighted gene coexpression network analysis (WGCNA) and pseudotime analysis to identify key genes. In addition, we predicted drug sensitivity relationships for key genes in CTD and predicted the correlation between drugs and survival through siGDC.

**Results:** We found that there were some CSCs in MIBC samples. The CSC population was heterogeneous during tumor development and was divided into quiescent and proliferating CSCs. We identified DBI as the key gene in quiescent CSCs. Analysis of a TCGA data set showed that higher DBI expression indicated higher histological grade. In addition, we predicted that acetaminophen can reduce DBI expression, thereby reducing the stemness of CSCs. Thus, we identified a potential new use of acetaminophen.

**Conclusion:** We systematically explored CSCs in tumors and determined that DBI may be a key gene and potential therapeutic target in quiescent CSCs. In addition, we confirmed that acetaminophen may be a candidate drug targeting CSCs, improving our understanding of CSC-targeting therapeutic strategies.

## Introduction

Muscle-invasive bladder cancer (MIBC) is an aggressive disease with high mortality and a propensity for metastatic dissemination (Bochner et al., 2006; Lenis et al., 2020). Neoadjuvant chemotherapy (NAC) followed by radical cystectomy is the standard of care for patients with MIBC (Bellmunt and Petrylak, 2012). However, even in patients who receive optimal treatment with surgery and chemotherapy, the 5-years overall survival rate is only 60% due to recurrence and metastasis (Soloway, 2013).

*Cancer* stem cells (CSCs) have received attention as a small population of highly malignant cells within liquid and solid tumors responsible for tumor initiation, self-renewal, chemo- and radiotherapeutic resistance, and evasion of immune surveillance to accelerate recurrence and metastasis (Magee et al., 2012; Nguyen et al., 2012; Visvader and Lindeman, 2012; Marquardt et al., 2018). Therefore, specific targeting of CSCs may improve the efficiency of cancer treatment (Du et al., 2019; Duan et al., 2021). However, CSCs constitute only a small fraction of the total tumor cell population (Reya et al., 2001), and a broad consensus on approaches to identify CSCs in clinical specimens is lacking. Furthermore, CSCs may be more heterogeneous than previously recognized, complicating their identification and eradication (Batlle and Clevers, 2017). Studies have shown that environmental signals can temporarily induce the proliferation of cells, but CSCs can reenter a quiescent state (Cai et al., 2017). However, many cell surface markers and signaling pathways differ between quiescent and proliferating CSCs. In addition, drug sensitivity and resistance may differ between quiescent and proliferating cells. Studies have shown that drugs therapeutically targeting the Wnt signaling pathway may eliminate proliferating CSCs but not quiescent CSCs (Choi et al., 2013). Unfortunately, quiescent CSCs have a strong ability for self-renewal, leading to tumor recurrence. Therefore, more attention should be devoted to quiescent CSCs, as the more recalcitrant subpopulation of CSCs.

Single-cell RNA sequencing (scRNA-seq) has been widely used to measure gene expression in individual cells. This approach can allow more comprehensive exploration of the tumor microenvironment and characterization of rare cells, thus overcoming the limitations of traditional bulk RNA sequencing (Li et al., 2012; Gawad et al., 2016; Plass et al., 2018). Through scRNA-seq, we can conduct a more accurate analysis to identify important pathways and genes related to CSCs. In previous studies, researchers found quiescent CSCs in tumors, which may be the cause of tumor recurrence and drug resistance, using scRNA-seq. These CSCs have lower proliferative activity and higher self-renewal ability than proliferating CSCs (Wang et al., 2021). Therefore, it is particularly important to define the molecular characteristics of quiescent CSCs and find targeted drugs.

Drug discovery and development is an expensive and time-consuming task. Currently, the FDA has approved only three targeted drugs (vismodegib, ivosidenib, and venetoclax) for CSCs (Clarke, 2019), but none of these target bladder CSCs. Repurposing of older drugs has become an alternative strategy to overcome the considerable costs and time required for drug development. Old drugs can be successfully used for new purposes because these compounds have been clinically tested in humans and have acceptable known side effects. Thus, some old drugs may be able to be reused to treat CSCs.

Here, we used scRNA-seq to demonstrate that diazepam binding inhibitor (DBI) is an important molecule in bladder CSCs that can maintain their stemness. In addition, we demonstrated that acetaminophen can reduce the expression of DBI and inhibit tumor proliferation. In our research, we revealed the importance of DBI in CSCs and identified a new use of acetaminophen to therapeutically target CSCs.

## Methods and Materials

### Acquisition, Quality Control, and Analysis of scRNA-Seq Data

The data used for our research were downloaded from an online public database and present no ethical issues. We obtained single-cell MIBC data from publicly available GEO data sets (GSE130001, GSE146137), and only human data were obtained from GSE146137 (Sfakianos et al., 2020; Wang et al., 2020). According to the original articles, all samples had been sorted to obtain the CD45-negative subpopulation. A total of four specimens and 7,979 cells were used for this study.

We used criteria for filtering the single cells to exclude low‐quality cells (<500 genes/cell and >15% mitochondrial genes). Based on the Seurat manual (Hao et al., 2020), gene expression levels were normalized and scaled for downstream analyses. The four samples were integrated by the canonical correlation analysis (CCA) method using 2000 anchors. A total of 2000 highly variable genes identified by the FindVariableFeatures function in the Seurat package were used for principal component analysis (PCA)-based dimensionality reduction with RunPCA. t-distributed stochastic neighbor embedding (t-SNE) was utilized to visualize single-cell clustering using principal components (PCs) 1 to 30.

### Identification of Cell Clusters and Prediction of Copy Number Variations

To identify the population of epithelial cells in each sample, we used SingleR (Aran et al., 2019), an R package designed to identify different cell types. We used the dataset function in the celldex package and call HumanPrimaryCellAtlasData as the reference database to infer the cell type of each single cell.

We extracted the count matrix of epithelial cells and used it for CNV prediction. InferCNV (https://github.com/broadinstitute/inferCNV), an available method to identify evidence of somatic large-scale chromosomal copy number alterations with reference to “normal cells”, was used to predict the CNVs in scRNA-seq data (Ortega et al., 2017). We used the following settings: cutoff = 0.1 (this value generally works well with 10X Genomics data), denoising = TRUE, and reference cells = endothelial cells, NK cells, and tissue stem cells. A heatmap showing the relative expression intensities across each chromosome was generated. We extracted cells with obvious CNVs and defined them as malignant epithelial cells.

### Calculation of the mRNA Expression-Based Stemness Index

An innovative one-class logistic regression (OCLR) machine learning algorithm was used to perform multiplatform analysis of the transcriptome, methylome and transcription factor binding sites of stem cells; the mRNAsi, an independent stemness index, was thus obtained (Malta et al., 2018). The OCLR machine learning algorithm is an effective method of quantifying the cancer stemness index using two independent indices. Calculation of stemness of tumor cells using the OCLR algorithm has been effectively applied in a variety of malignant tumors. We identified one cluster of 279 cells with the highest mRNAsi values as the CSC cluster. The stemness index was calculated via the OCLR algorithm by utilizing the scCancer (version 2.2.1) package in R (Guo et al., 2020).

### Pseudotime Analysis

Monocle two was applied to generate single-cell pseudotime trajectories from scRNA-seq data assuming that the one-dimensional quantity “time” can describe high-dimensional expression values. Briefly, we selected genes that were differentially expressed (*p*-value < 0.05) among t-SNE clusters as candidate genes. And converted the Seurat object into CellDataSet through setOrderingFilter, and estimated the size factor and dispersion. Set the dimensionality reduction parameter in reduceDimension to “DDRTree” to perform dimensionality reduction for all epithelial cells. And through the orderCells function, cell sorting and trajectory construction are performed.

### Weighted Gene Coexpression Network Analysis

We selected 279 cells from the CSC cluster to construct a gene expression matrix for WGCNA (Zhang and Horvath, 2005; Langfelder and Horvath, 2008). A signed network was constructed using 2000 genes selected with the FindVariableFeatures function in the Seurat package. After constructing the adjacency matrix, we chose the soft-thresholding power (*β* = 3) to obtain the topological overlap matrix (TOM). Genes were grouped using average linkage hierarchical clustering based on the high similarity of coexpression relationships.

### pySCENIC

Single-cell regulatory network inference and clustering (SCENIC) is a new computational method to map genes regulatory networks and identify stable cell states by evaluating the activity of each cell from scRNA-seq data (Aibar et al., 2017). pySCENIC was performed on all cells, and the regulons were calculated based on transcription factors (TFs) or their target genes. Only regulons significantly upregulated or downregulated were involved in further analysis.

### Gene Ontology Analysis and Single-Sample Gene Set Enrichment Analysis

ClusterProfiler (version 3.18.1) was applied to analyze differences among clusters (Yu et al., 2012). Based on the GO database, functional enrichment analysis of marker genes (log2FC ≥ 1, adjusted *p* ≤ 0.05) was performed on each cell to explore their potential biological functions. ssGSEA was applied to calculate the relationship between the stemness index of each cell in the CSC cluster and the “Hallmark” gene sets (version 7.3) using the R package GSVA (1.38.2) with the “ssgsea” option for the method argument (Hänzelmann et al., 2013).

### Drug Selection

The Comparative Toxicogenomics Database (CTD, http://ctd.mdibl.org/) is a public resource that constructs chemical-gene-disease networks and predicts novel relationships using different data. This database facilitates the generation of testable hypotheses about the molecular mechanisms linking drugs and genes (Davis et al., 2011). Combining the search results with the relevant literature reports, we selected acetaminophen as the target drug for CSC therapy.

Survival interaction of Genes, Cells and Drugs in human cancers (siGCD, http://sigcd.idrug.net.cn) is a web server for exploration of the interactions of genes, cells and drugs with survival in human cancers. We predicted the correlation between acetaminophen and survival by using the “Cell & Drug” module in this database. We defined “Cell” as “*Cancer* stem cell-Bladder” and “Drug” as “acetaminophen”.

## Results

### Single-Cell Analysis Identified Cellular Subtypes and Inferred Malignant Epithelial Cells

**FIGURE 1:**
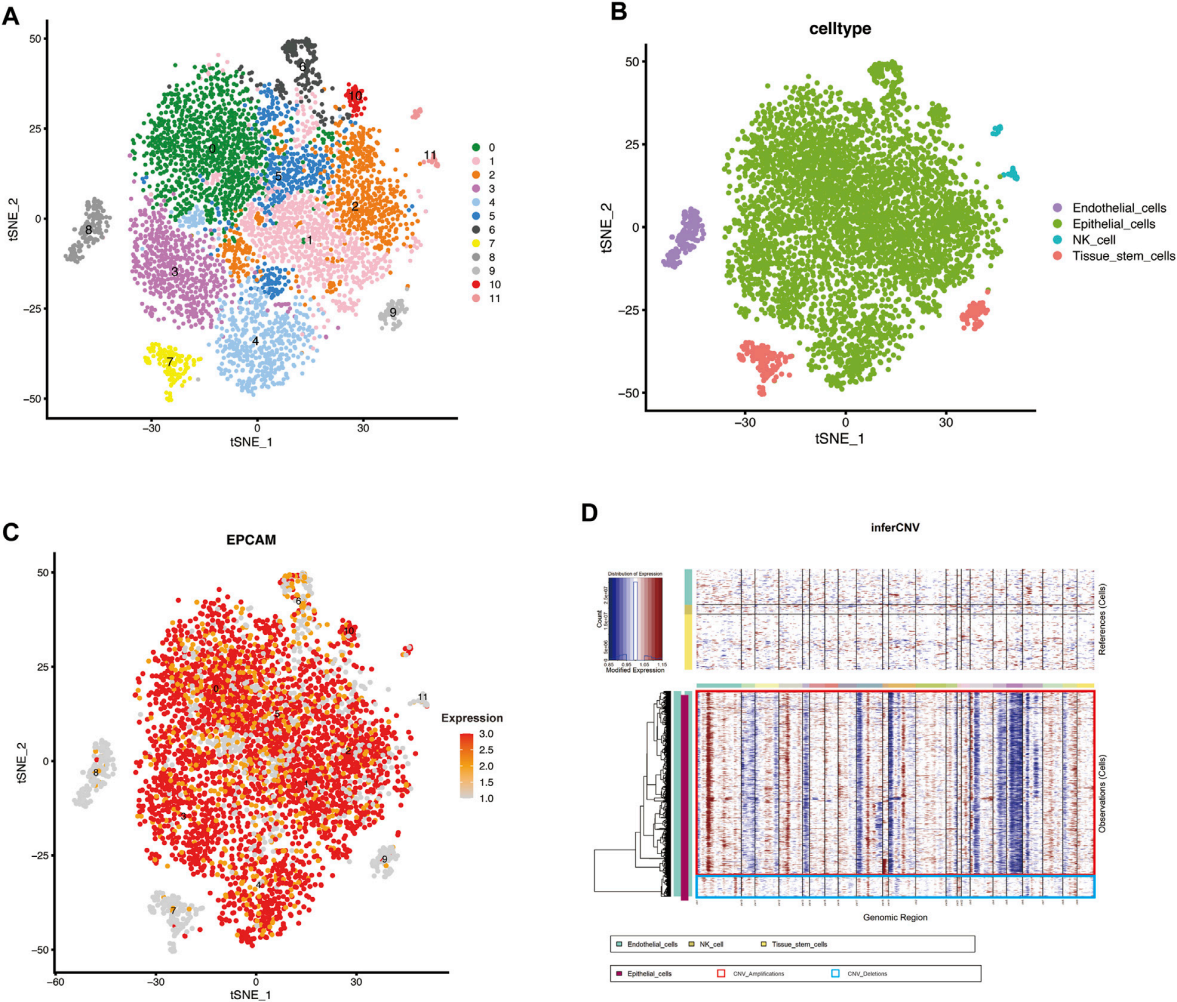
scRNA-seq identified the cellular components in MIBC and inferred malignant epithelial cells **(A,B)** t-SNE plot showing clustering information in MIBC **(C)** FeaturePlot was used to demonstrate the identity of epithelial cells through the expression of a well-known specific marker (EPCAM) **(D)** Heatmap showing the chromosomal landscape of inferred large-scale copy number variations (inferCNVs) distinguishing individual tumor (malignant) cells from nontumor cells. Red box: Amplifications of CNV, Blue box: Deletions of CNV.

The scRNA-seq data were screened with strict QC criteria (Supplementary Figure S1A), and 7,228 cells were obtained for follow-up analysis after QC. To determine the components of tumor cells, we first set the resolution at 0.5, resulting in the identification of 12 cell clusters, and visualized the clusters utilizing a t-SNE plot (Figure 1A).

To identify different genes in each cluster, we used the FindAllMarkers function in Seurat to obtain the key genes in each cluster. The ten genes with the highest expression levels in each cluster are shown in the heatmap (Supplementary Figure S1B). Based on the expression of the key genes, the 12 cell clusters were divided into four different cell types (Figure 1B), which were identified with the SingleR (version 1.4.1) R package. Because the included data were only from cells without expression of CD45, which is recognized as the marker gene of lymphocytes (Clement, 1992), lymphocytes were almost completely absent from this analysis. The common epithelial cell marker EPCAM was expressed in the “Epithelial_cells” subpopulation, verifying the accuracy of the identification (Figure 1C). Finally, we extracted the cells defined as “Epithelial_cells” for subsequent analysis.

To reveal the changes in epithelial cells during malignant progression of MIBC, we estimated the CNV of epithelial cells with inferCNV (version 1.6.0) and set the reference group to nonmalignant cells (endothelial cells, NK cells, and tissue stem cells) (Xiong et al., 2020; Rindler et al., 2021; Wu et al., 2021). We clustered cells according to the degree of CNV; blue indicates deletions, and red indicates amplifications (Figure 1D). In Figure 1D, the epithelial cells in the red box have a significantly higher CNV degree than the cells in the blue box. Finally, the cells in the red box were extracted and defined as malignant epithelial cells.

### Stemness of Malignant Epithelial Cells in Each Cell Cluster Based on mRNAsi

**FIGURE 2:**
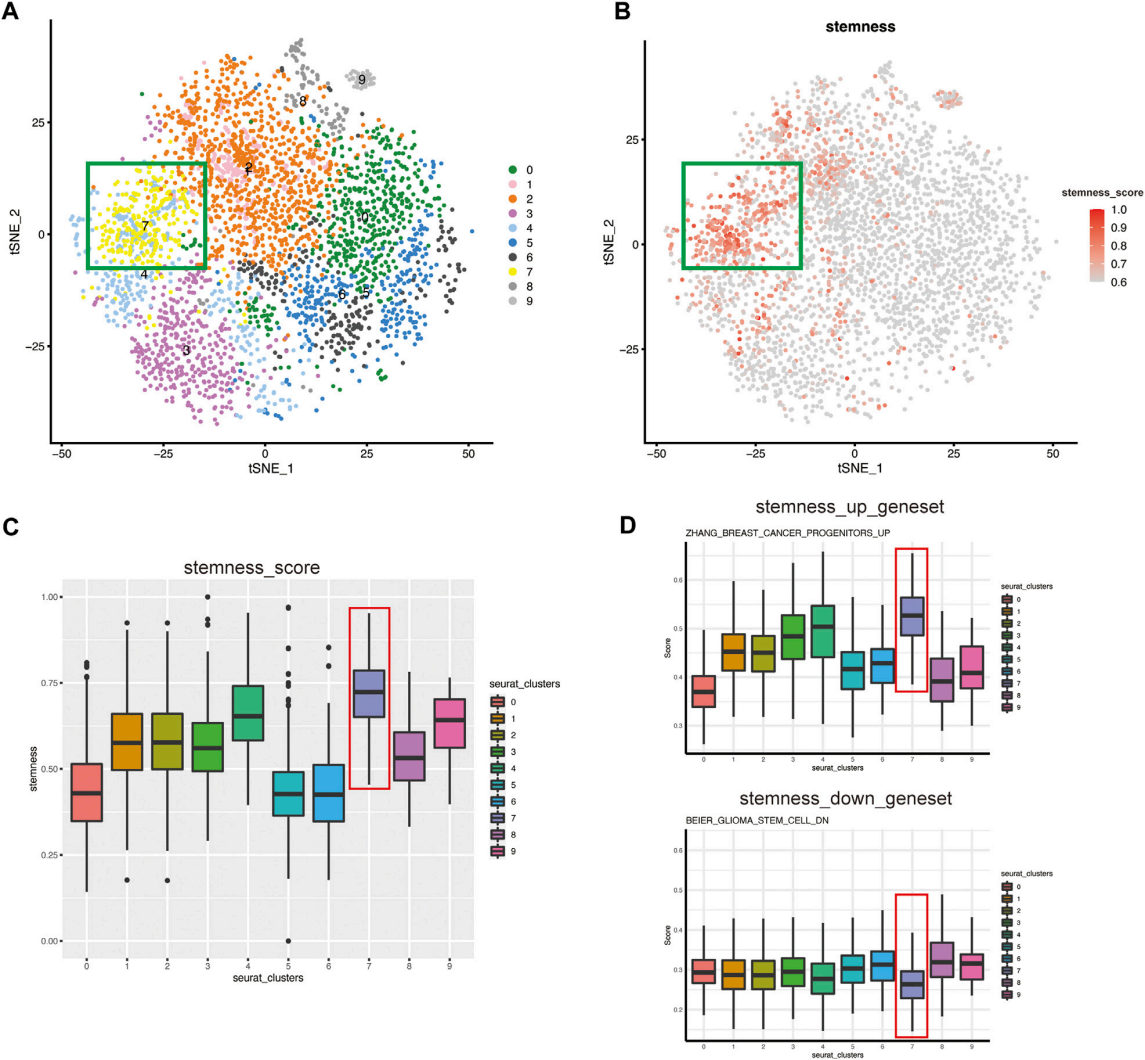
The CSC subgroup was identified and verified by evaluation of stemness **(A)** t-SNE plot of malignant epithelial cells isolated across all specimens, colored and labeled by cluster. The cells of clusters-7 in the green box have higher stemness **(B)** t-SNE plot showing the stemness score for each cell. The color of the dots represents the stemness, and the darker the color, the higher the stemness. The cells in the green box have the highest stemness **(C)** Box plot showing the stemness score for each cluster. Cluster-7 has the highest stemness **(D)** ssGSEA score of CSCs-related gene set in each seurat_clusters. Top: Represent the gene set of stem genes upregulation. Bottom: Represent the gene set of stem genes downregulation.

We reclustered the malignant cells with a resolution of 0.8 and obtained ten clusters (Figure 2A). The t-SNE plot shows that the tumor epithelial cells formed distinct clusters, indicating that the gene expression pattern of epithelial cells gradually changes during the progression of MIBC. To detect CSCs, we used the OCLR model (Malta et al., 2018) to estimate the mRNAsi of each individual tumor cell and found that the cells of clusters-7 in the green box have higher stemness than other clusters (Figure 2B). The box plot intuitively shows that the stemness of cluster-7 was higher than that of the other clusters (Figure 2C).

We performed ssGSEA to verify the features of cell stemness. “Hallmark” gene sets were significantly enriched, and the results showed that as stemness increased, hallmark gene sets such as DNA_REPAIR (Zhang et al., 2017), MYC_TARGET (Hanahan and Weinberg, 2011; Bahr et al., 2018), and OXIDATIVE_PHOSPHORYLATION were activated (Supplementary Figure S1F). These results were consistent with the enrichment results in the original article and suggested the strong self-renewal ability of CSCs (Malta et al., 2018). To further identify the CSC-like cluster, we selected CSCs-related up-regulated gene sets and down-regulated gene sets from MsigDB for evaluation of all clusters. And the results showed that, cluster-7 had a higher score in the gene set of stem genes upregulation, while lower scores in the gene set of stem genes downregulation (Figure 2D). It further confirmed that cluster-7 had the highest stemness. Therefore, we tentatively defined cluster-7 as a CSC-like cluster.

### A Stemness Gene Set in CSCs Was Identified by WGCNA

**FIGURE 3:**
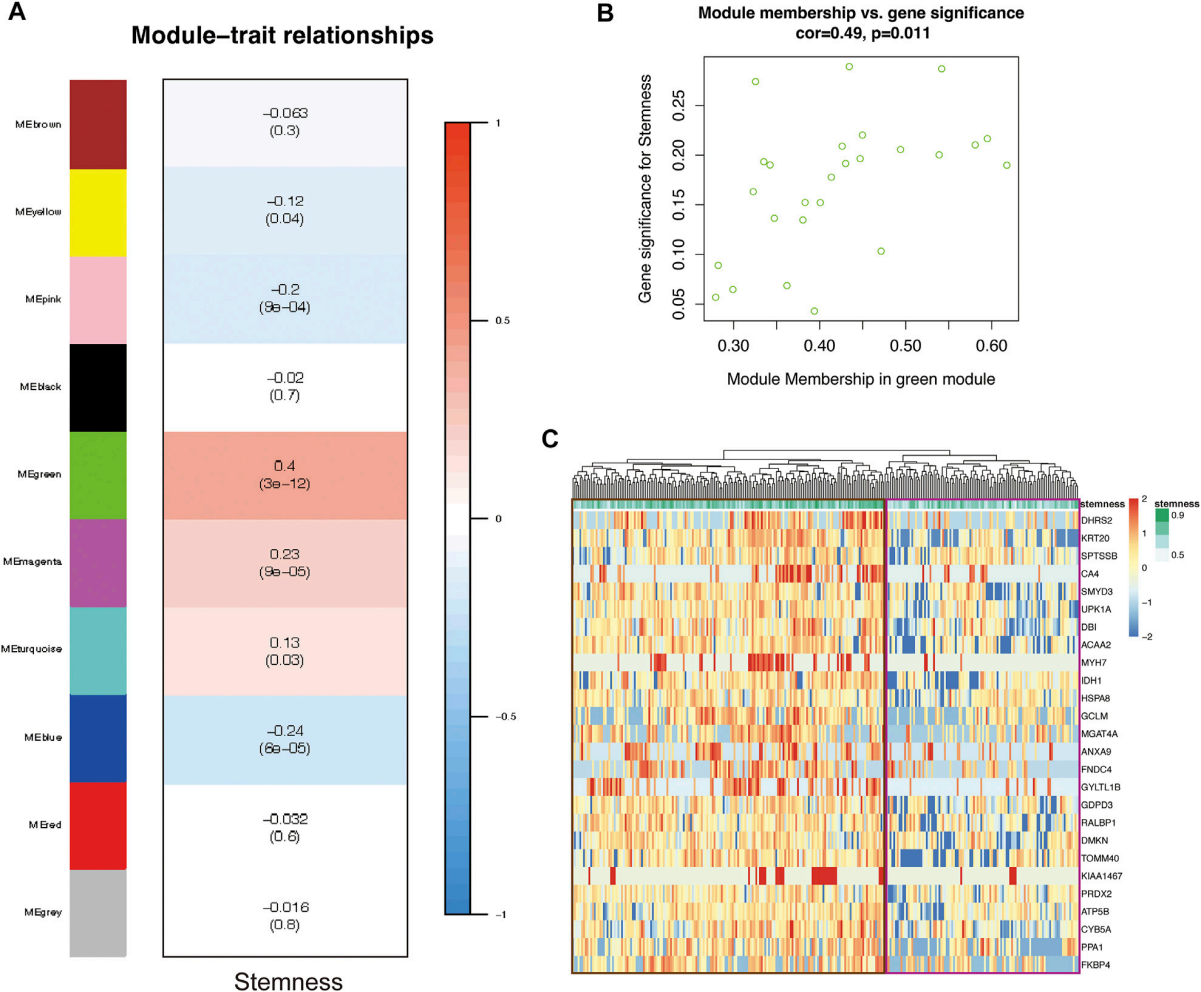
The CSC gene-network module was identified by WGCN A. **(A)** Heatmap showing module associations. Each row corresponds to a module eigengene; each column, to stemness. In addition, each box contains the corresponding correlation and *p*-value **(B)** Scatter plot of gene significance (GS) for CSCs vs. module membership (MM) in the MEgreen module. There is a significant correlation between CSCs and the MEgreen module **(C)** Heatmap showing the relationship between 26 genes in the MEgreen module and stemness (cells with higher stemness are in the brown box; cells with lower stemness are in the purple box).

WGCNA is a systems biology method for describing correlation patterns among genes in RNA sequencing data (Zhang and Horvath, 2005; Langfelder and Horvath, 2008). We exploited this powerful tool to identify the hub genes that induce the stemness of tumor cells. Then, we extracted 279 cells in cluster-7 for WGCNA. After we chose the soft-thresholding power (*β* = 3), the algorithm for gene network construction and module identification was run, and 10 modules were identified (Figure 3A). There was a significant correlation between the MEgreen module and the stemness of cells (Figure 3A). To better prove the correlation between gene significance for CSCs and module membership of the MEgreen module, a scatter plot was generated (Figure 3B). These results indicated that the MEgreen module could accurately represent the CSC subpopulation.

The MEgreen module contained 26 genes. To further support the correlation between the expression of these genes and cell stemness, we generated a Heatmap to visualize the relationship (Figure 3C). The cells in the brown box had higher stemness, and the expression of these genes in these cells was significantly higher than that in the cells in the purple box (which had lower stemness). This pattern proved that high expression of these genes was positively correlated with enhanced stemness of CSCs.

### pySCENIC Analysis Revealed Abnormally Activated Transcription Factors in CSCs

**FIGURE 4:**
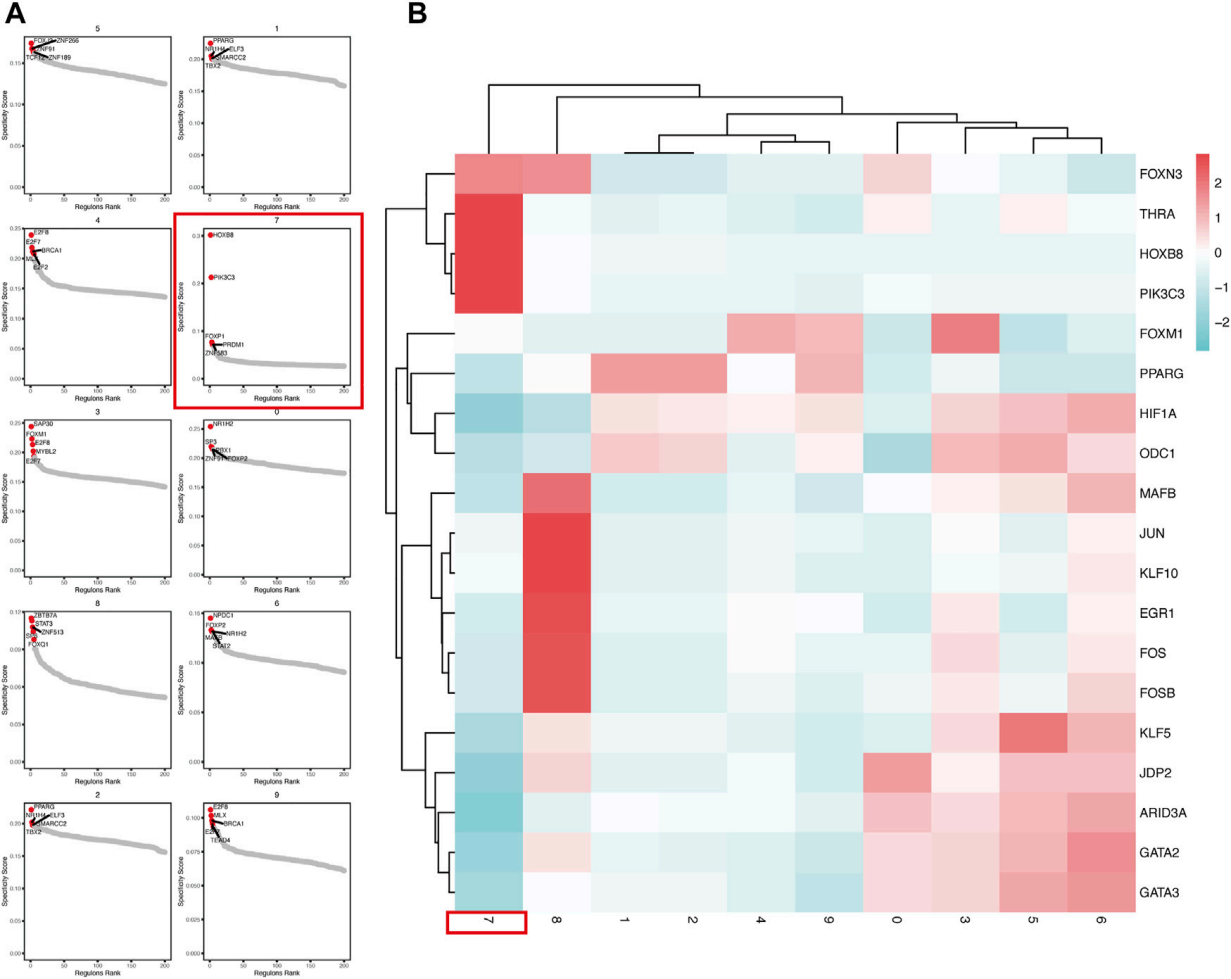
pySCENIC analysis revealed abnormally activated transcription factors in CSCs **(A)** Top5 transcription factor abnormally activated in each tumor cell cluster **(B)** The Heatmap showed the activation of transcription factors activity in different tumor cell clusters.

In order to explore the transcriptional regulation inside the CSCs cluster (cluster-7), we then used pySCENIC to infer transcription factors (TFs) regulatory information underlying each cluster. SCENIC analysis revealed that some TFs had obvious differential activation in the CSC. We then compared and detected five up-regulated TFs in each cluster (Figure 4A).

In the CSCs cluster (cluster-7), we can clearly see that the PIK3C3, HOXB8 and THRA are highly activated (Figure 4B). The Class III phosphoinositide 3-kinase (PIK3C3), also known as vacuolar protein sorting 34 (Vps34), plays an important role in the control of autophagy, which are critical in a wide range of cellular processes. The suppression of PIK3C3 inhibits the activation of SGK3, a CSCs promoter induced by PI3K inhibitors. And PIK3C3 inhibitors inhibit liver CSCs by activating AMP-activated kinase (AMPK) (Liu et al., 2020). HOXB8 has been shown to be associated with the development of various cancers, such as colorectal, hepatocellular and gastric cancers (Kanai et al., 2010; Ding et al., 2017). *In vitro* studies by Vider et al. showed that HOXB8 is upregulated in colorectal cancer cell lines. Furthermore, upregulated expression of HOXB8 was observed in all stages of colorectal cancer (Vider et al., 2000). At the same time, HOXB8 also upregulates STAT3 expression, which induces the development of EMT. In addition, the activation of the miR-133b/HOXB8 axis also promotes tumor stemness, proliferation and invasion (Jiang et al., 2020).

### Pseudotime Analysis Identified the Quiescent CSCs

**FIGURE 5:**
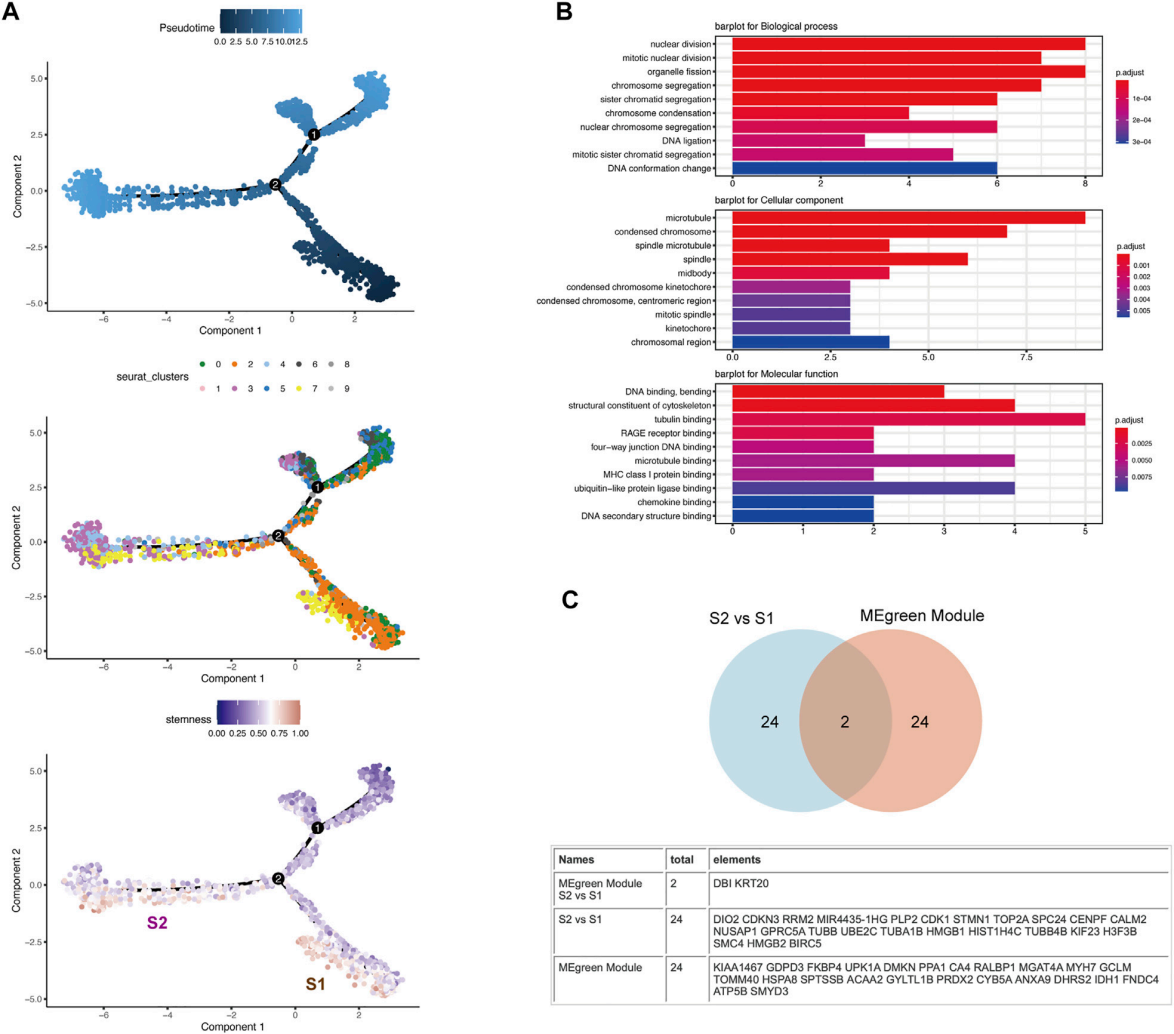
Pseudotime analysis revealed the heterogeneity of CSCs along their developmental trajectory **(A)** The distribution of pseudotime (top), cluster (middle) and stemness score (bottom) exhibits a continuous pattern. Top: Pseudotime is shown colored along a gradient from dark to light blue, and the start of pseudotime is indicated. Middle: Clusters are color-coded by subpopulation. Bottom: CSCs are divided into two subsets (S1 and S2) along their developmental trajectory **(B)** The figure shows that the differentially expressed genes with higher expression in S2 than S1 were extracted and screened for GO analysis. The results of the GO analysis suggests that the S2 subgroup shows higher division and proliferation abilities than the S1 subgroup **(C)** Venn diagram depicting the intersection between the DEGs of S2 vs. S1 and the gene set corresponding to the MEgreen module in WGCNA.

To better understand the functional states and relationship of CSCs, we next determined their developmental trajectories with Monocle 2 (Supplementary Table S4). Pseudotime analysis showed that the cells in cluster-7 were divided into early and late groups on the time trajectory (Figure 5A). The differentially expressed genes (DEGs) with higher expression in S1 than S2 were DBI and KRT20, and the rest of the DEGs were highly expressed in S2. We extracted the highly expressed DEGs in S2 for GO functional enrichment analysis to compare the late cluster-7 cell group (S2) with the early cluster-7 cell group (S1), and we found that the S2 cell group were involved in more functions, such as nuclear division and chromosomal division (Figure 5B). This result suggested that S2 cells have high proliferative activity and might be a group of cells that increase tumor malignancy. In contrast, the S1 cells were in a relatively quiescent state. We defined the S1 cells as “quiescent CSCs” and S2 cells as “proliferating CSCs” (Clarke, 2019). Furthermore, tumor recurrence and metastasis are closely related to quiescent CSCs (Wang et al., 2021). Therefore, we identified the differentially expressed genes (DEGs) between the quiescent CSCs and proliferating CSCs (Supplementary Table S1).

By comparing the gene set corresponding to the MEgreen module in WGCNA and the DEGs between S2 and S1, we found that DBI and KRT20, which are closely related to stemness, appeared in both gene sets (Figure 5C). By searching the CellMarker database (http://biocc.hrbmu.edu.cn/CellMarker/), we found that DBI and KRT20 are closely related to embryonic cells, fetal cells, and CSCs (Supplementary Table S2). We evaluated the correlations among KRT20, DBI and common CSC markers (PROM1/CD133 (Aghajani et al., 2019) and CD24 (Ooki et al., 2018)) in the TCGA database and found that the expression of these molecules showed a trend toward a positive correlation (Supplementary Figure S1C). And with the progress of the pseudotime, the expression of DBI and KRT20 shows a downward trend (Supplementary Figure S1D). Meanwhile, we evaluated the correlation of DBI and KRT20 expression with cell stemness and plotted the scatter plot (Supplementary Figure S1E).DBI, KRT20 and cell stemness were all positively correlated, with the strongest correlation between DBI and stemness (R = 0.37, *p* < 2.2e-16). Therefore, we deemed DBI and KRT20 to be closely associated with quiescent CSCs, and DBI as a follow-up study.

### Acetaminophen Decreased DBI Expression and Inhibited Tumor Proliferation

**FIGURE 6:**
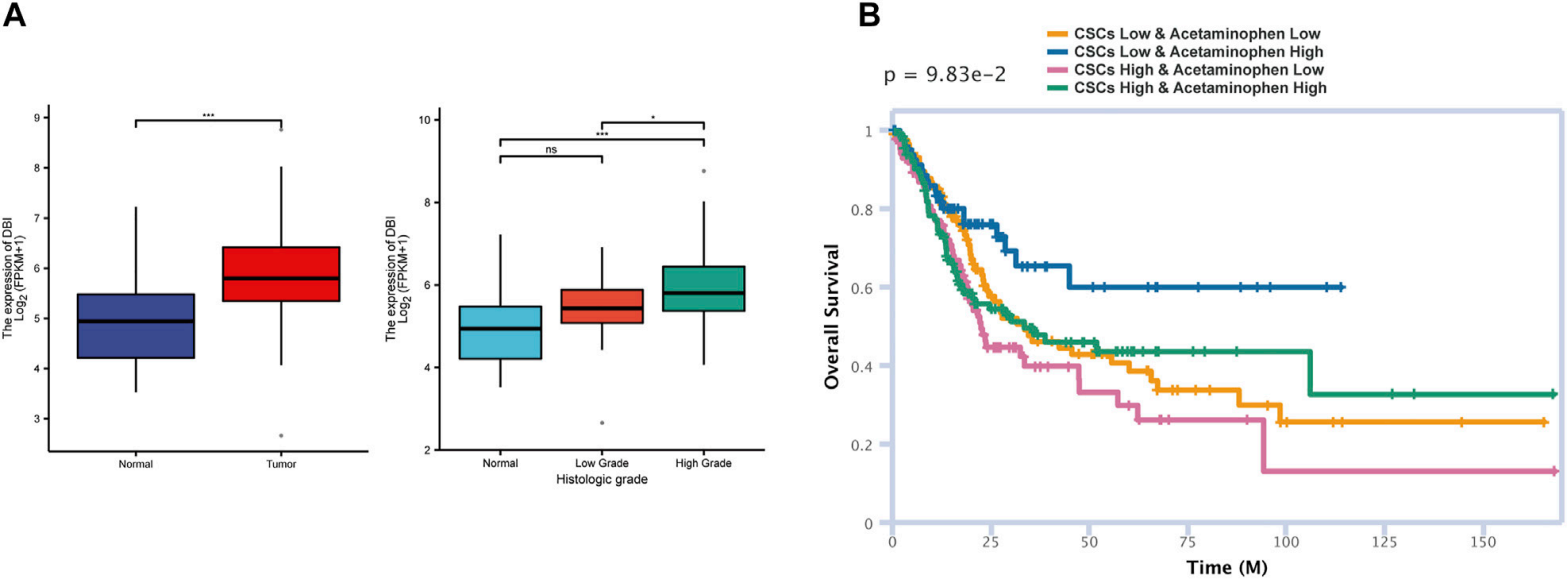
Molecule and drug prediction in the database (TCGA) **(A)** Analysis of the TCGA BLCA data of matched tumor–normal tissue pairs showing that DBI expression is higher in tumor tissue than in normal tissue (left). The higher the DBI expression, the higher is the histological grade (right) (*p < 0.05; **p < 0.001) **(B)** The siGCD database was used to predict the effects of acetaminophen and CSCs on patient survival.

As a marker of bladder cancer CSCs newly discovered in our research, DBI has rarely been reported in previous articles. Higher stemness is related to a higher degree of active tumor dedifferentiation, suggesting that stemness is related to histopathological grade (Malta et al., 2018). We analyzed the relationship between DBI expression and clinical histological grade in the TCGA database and found that the higher the expression of DBI, the higher was the histological grade (Figure 6A), which means that DBI may enhance the stemness of CSCs, leading to increased tumor heterogeneity (Reddy, 2020; Zeng et al., 2021). Therefore, we focused on DBI molecules in our research.

Through the CTD database, we screened targeted drugs for interactions with DBI. Among the candidate drugs and compounds, acetaminophen can reduce the expression of DBI (Supplementary Table S3). Acetaminophen and its metabolites have been proven to kill CSCs in previous studies (Ahmed et al., 2019; Pingali et al., 2021). Then, we used the siGCD database to predict the relationships among drugs, cells and survival, defining “Cell” as “*Cancer* stem cell-Bladder” and “Drug” as “acetaminophen”. The prediction results indicated that under the same dose of acetaminophen, a higher CSCs count indicates a poorer prognosis (Figure 6B), which is closely related to tumor recurrence and drug resistance mediated by CSCs (Bayik and Lathia, 2021). Moreover, a high dose of acetaminophen predicted a good prognosis in both the high and low CSC groups, suggesting that acetaminophen exerts great therapeutic effects against CSCs. The above analysis results indicated that acetaminophen may kill CSCs by inhibiting the expression of DBI, thereby inhibiting tumor growth and recurrence. We speculate that acetaminophen may be a candidate for the treatment of quiescent CSCs.

## Discussion

Drug resistance and recurrence of bladder cancer, which strongly affect the therapeutic efficacy and survival rate of patients, are highly correlated with CSCs. However, CSCs are rare cells with stem cell characteristics within the tumor cell population, accounting for only a small proportion of the total cell population in tumor tissue. Therefore, accurately sorting and killing CSCs is difficult. It is difficult to identify and sort CSCs from traditional bulk RNA sequencing data because the cell population is mixed. scRNA-seq overcomes this limitation and has become one of the most important tools for studying CSCs. Therefore, we analyzed CSCs using scRNA-seq and found that DBI is an important prognostic marker and potential target for quiescent CSCs. In addition, our data suggested that acetaminophen can reduce the stemness of CSCs by reducing the expression of DBI.

Clinical translational research on CSCs has bright prospects but is only in its infancy. Several problems need to be solved before the clinical potential of CSCs can be realized (Clarke, 2019). First and foremost, it is necessary to further define the molecular and cytological characteristics of CSCs. Obviously, not all carcinogenic mutations can pass stemness to CSCs. Second, therapeutic drugs should be able to target both quiescent and proliferating CSCs. By targeting the microenvironment of CSCs, drugs abolish the self-renewal ability of CSCs, thus eliminating the root causes of tumor development. In this study, we isolated the CSC population by evaluating the stemness of each cell and identified a stemness gene set through WGCNA. Then, this CSC population was divided into two subsets, S1 and S2, by pseudotime analysis, and enrichment analysis suggested that S1 was quiescent CSCs and S2 was proliferating CSCs. Studies have shown that the CSC population is heterogeneous during tumor development. A small subset of CSCs, called quiescent CSCs, have a greater ability for self-renewal than for proliferation. These cells are less sensitive to treatment and maintain the renewal of tumor cells, leading to tumor recurrence. DBI was highly expressed in quiescent CSCs and was highly correlated with the stemness gene set in WGCNA. To verify this result, we used the TCGA database to increase the number of samples. DBI expression was significantly higher in tumors; moreover, higher expression of DBI suggested higher histopathological grade (Figure 6A). This finding also suggests that DBI may maintain the stemness of CSCs, thereby promoting tumor heterogeneity.

DBI (also named acyl-CoA-binding protein, ACBP) is a 10-kD protein that binds, buffers and transports acyl-coenzyme A molecules, contributing to lipid metabolism (Knudsen et al., 1993; Lebrun et al., 2021). A previous study showed that this metabolic reprogramming mediated by DBI can promote tumor development (Duman et al., 2019). Interestingly, several studies have shown that DBI can maintain the proliferation of stem and progenitor cells (Alfonso et al., 2012; Dumitru et al., 2017). Interestingly, DBI expression is not restricted to gliomas but is also present in most tumor types pointing to the possibility of a general mechanism of DBI in other cancers. Indeed, in non-small cell lung cancer, DBI has been shown to promote tumor cell proliferation *in vitro* by regulating FAO, and high DBI expression predicts a poorer prognosis for lung cancer patients (Harris et al., 2014). Considering these results collectively with our findings, we believe that DBI, as an important molecule for stemness maintenance, can serve as both a marker and therapeutic target for quiescent CSCs.

We speculated that acetaminophen can reduce the expression of DBI, suggesting that acetaminophen may be an important candidate for CSC treatment. In previous studies, acetaminophen has been shown effective in treating recurrent tumors and CSCs (Wu et al., 2013; Ahmed et al., 2019; Pingali et al., 2021). We thus believe that acetaminophen can be used as a DBI-targeting drug to reduce the CSC population, hence suppressing tumor recurrence and the development of drug resistance.

Our future research will address the following topics: 1) The specific mechanism by which DBI maintains CSCs needs further clarification by multiomics analysis, which will facilitate our understanding of CSCs; 2) The possible mechanisms by which DBI is inhibited by acetaminophen need verification through additional molecular biology experiments, which will improve treatments against CSCs; and 3) The possibility of combining acetaminophen with antidotes to combat its considerable side effects when used at high doses needs further exploration.

In summary, we demonstrated through scRNA-seq that quiescent CSCs are maintained by DBI and revealed that acetaminophen can be used as an inhibitor of DBI and CSCs. These findings are highly clinically relevant and provide a theoretical basis for a new therapeutic approach of combining acetaminophen with other drugs to treat incurable and recurrent tumors.

## Data Availability

The datasets presented in this study can be found in online repositories. The names of the repository/repositories and accession number(s) can be found in the article/Supplementary Material.
