# Supplementary figures and images for: Single-Cell Sequencing Reveals that DBI is the Key Gene and Potential Therapeutic Target in Quiescent Bladder Cancer Stem Cells
Source: Front Genet. 2022 Jun 3;13:904536. doi: 10.3389/fgene.2022.904536 (PMC9235029; doi:10.3389/fgene.2022.904536)

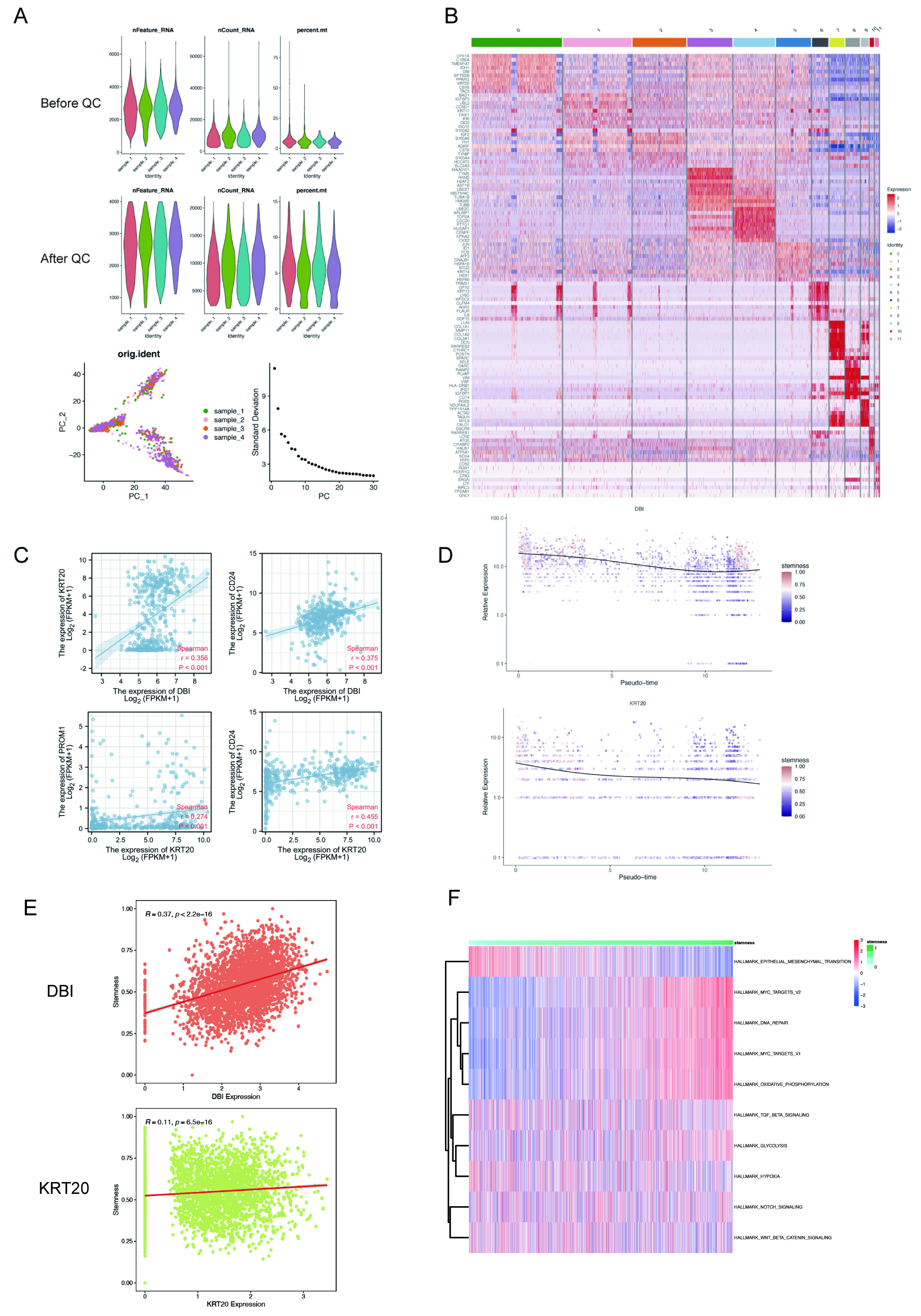

Supplement: Supplementary file 3 [file Image1.TIF]
